# Supplementary figures and images for: A caspase–RhoGEF axis contributes to the cell size threshold for apoptotic death in developing Caenorhabditis elegans
Source: PLoS Biol. 2022 Oct 6;20(10):e3001786. doi: 10.1371/journal.pbio.3001786 (PMC9536578; doi:10.1371/journal.pbio.3001786)

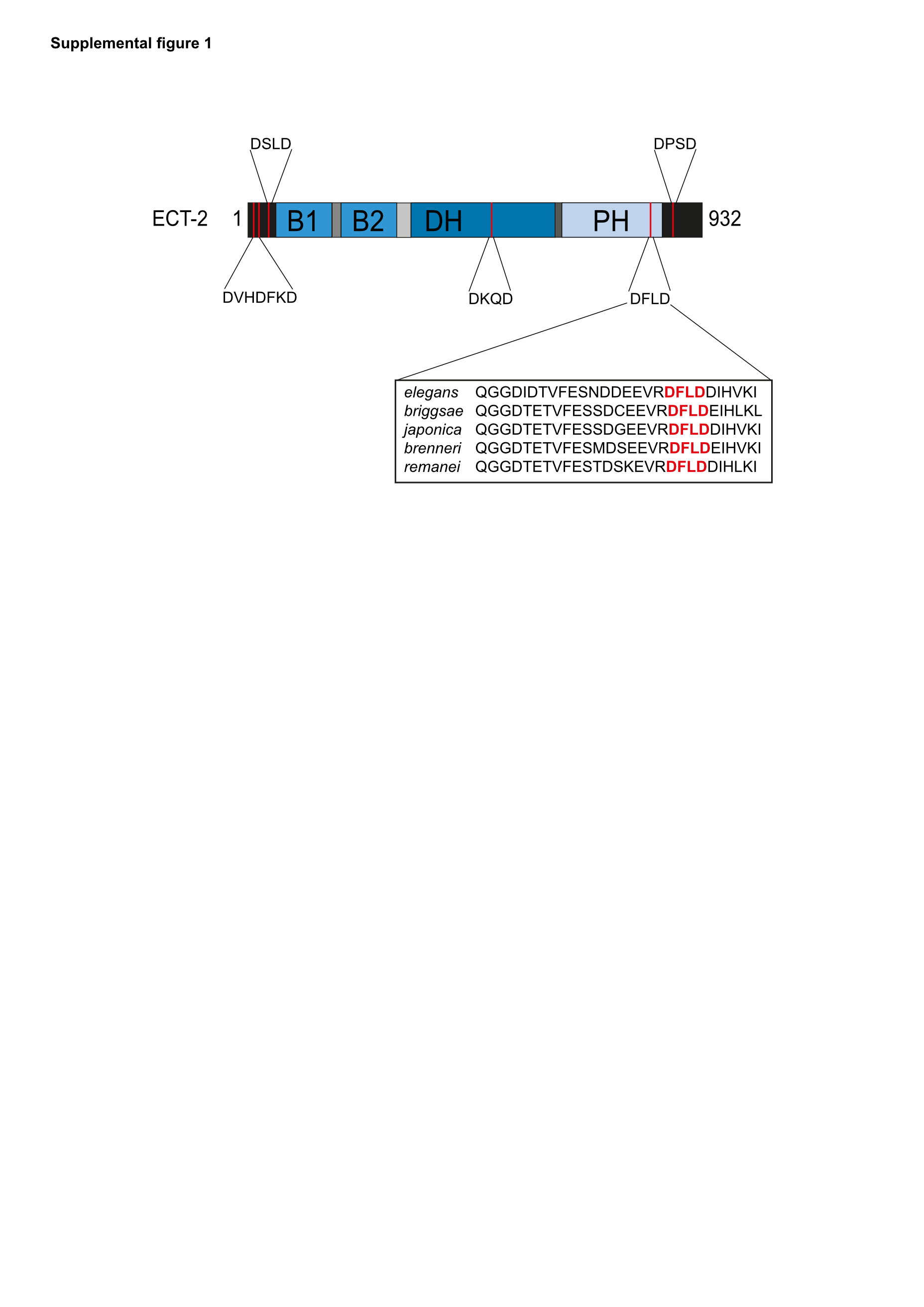

Supplement: S1 Fig — (TIF) [file pbio.3001786.s001.tif]

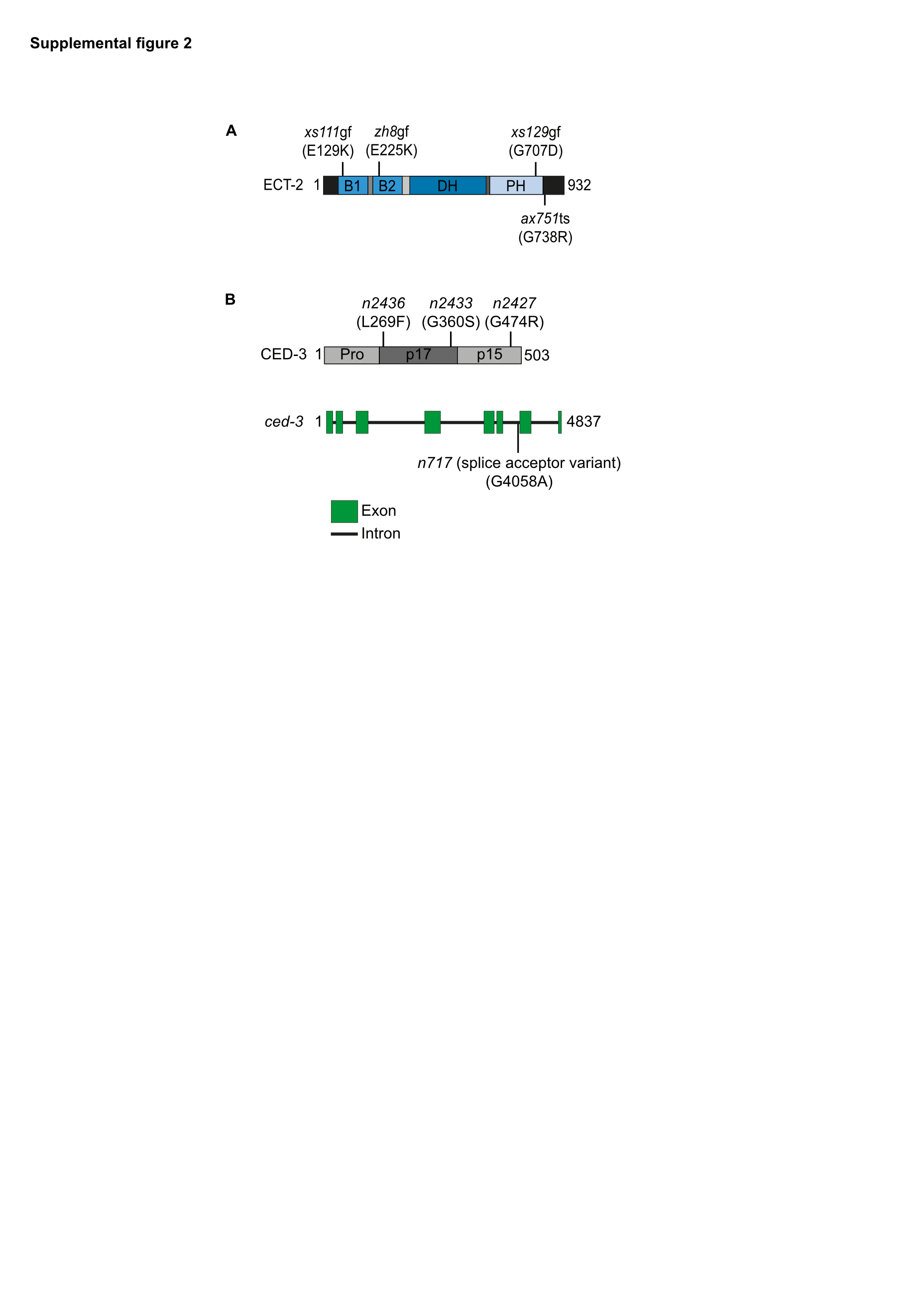

Supplement: S2 Fig — (TIF) [file pbio.3001786.s002.tif]

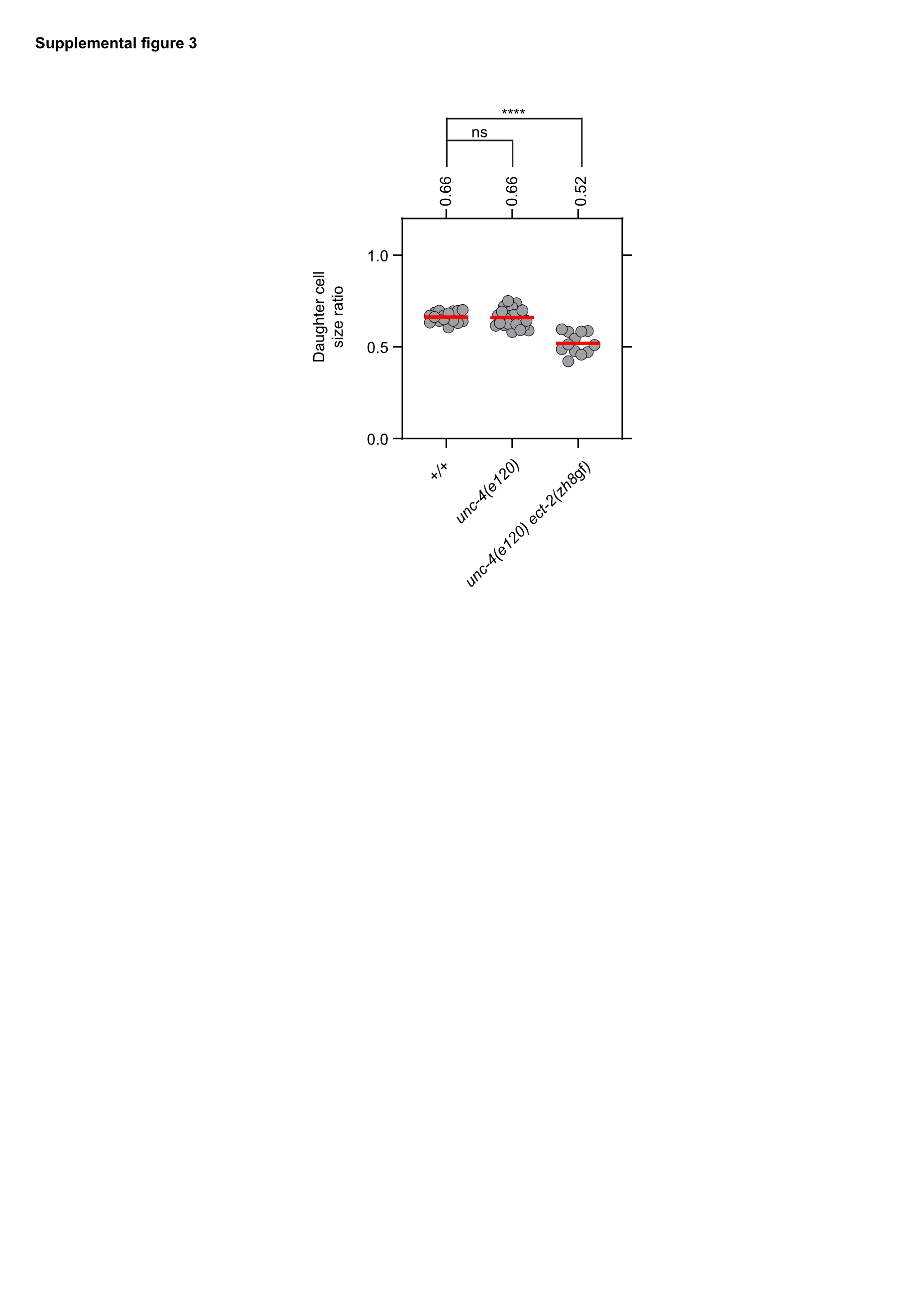

Supplement: S3 Fig — Each gray dot represents the daughter cell size ratio of 1 pair of daughter cells. The mean values are indicated using the horizontal red lines and are also provided on top. Statistical significance was determined using the Dunnett’s T3 multiple comparisons test (**** = P < 0.0001, ns = P > 0.05). (TIF) [file pbio.3001786.s003.tif]

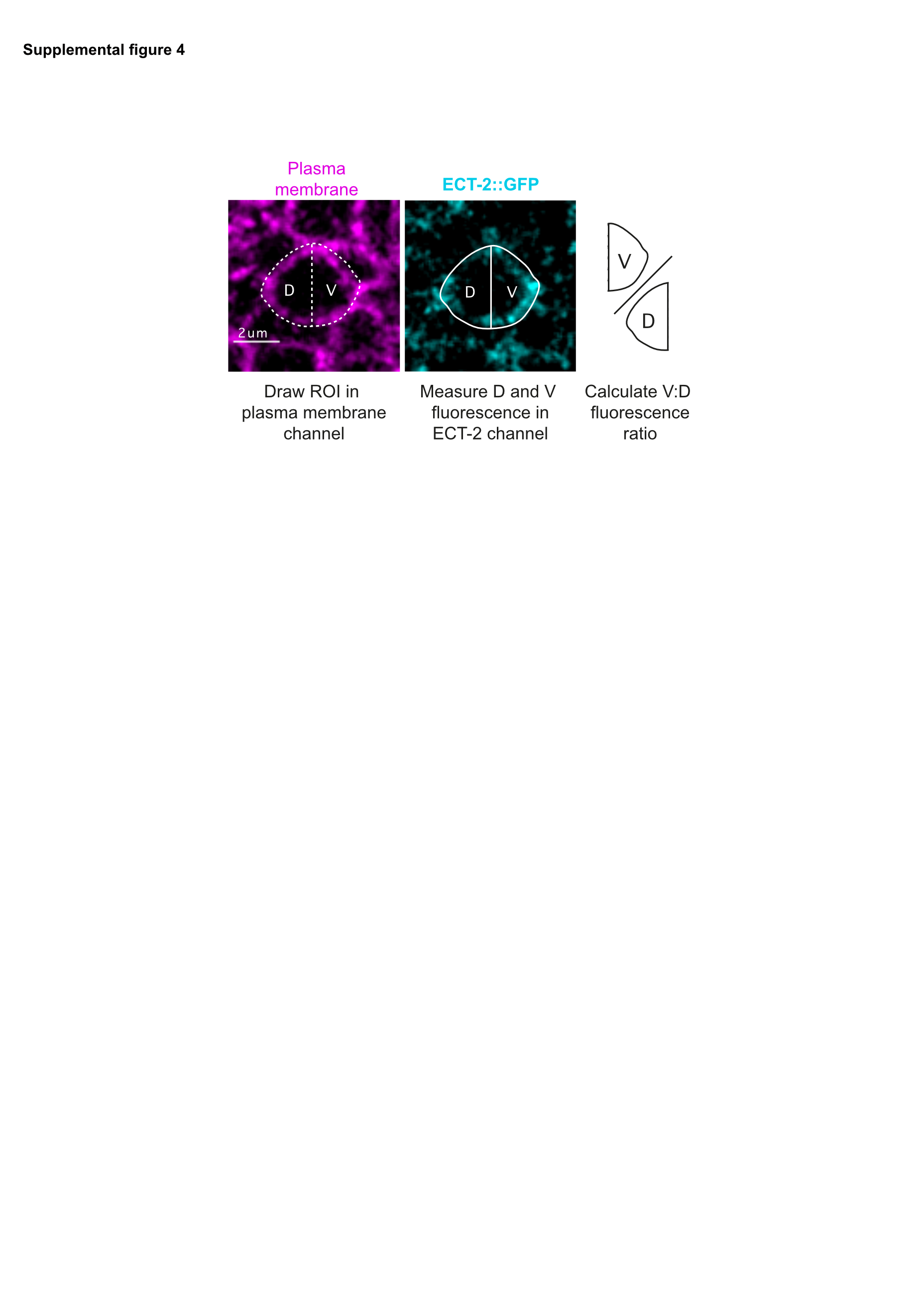

Supplement: S4 Fig — The plasma membrane of the NSM neuroblast was marked using the transgene ltIs44 (Ppie-1::mCherry::PHPLCΔ). The NSM neuroblast was divided into dorsal and ventral halves by drawing a vertical line along the center of the neuroblast. The mean fluorescence intensity of the appropriate transgene was measured in each half and divided to obtain their ratio. D is the dorsal side and V is the ventral side. Scale bar: 2 μm. (TIF) [file pbio.3001786.s004.tif]

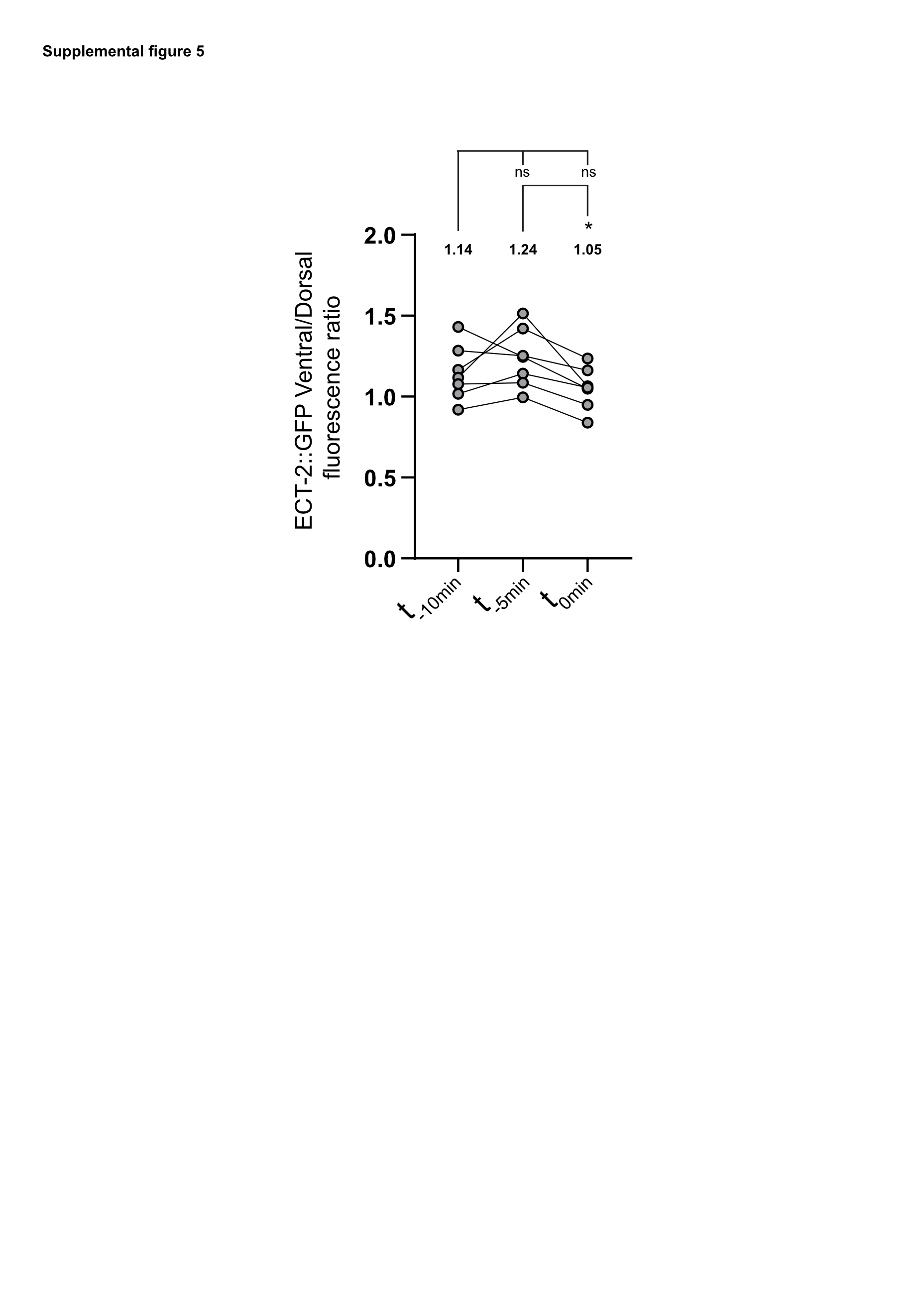

Supplement: S5 Fig — Each gray dot represents the ventral/dorsal fluorescence intensity ratio of 1 NSM neuroblast. The mean values are given on top. Statistical significance was determined using the Welch’s 2 sample t test (* = P < 0.05, ns = P > 0.05). (TIF) [file pbio.3001786.s005.tif]

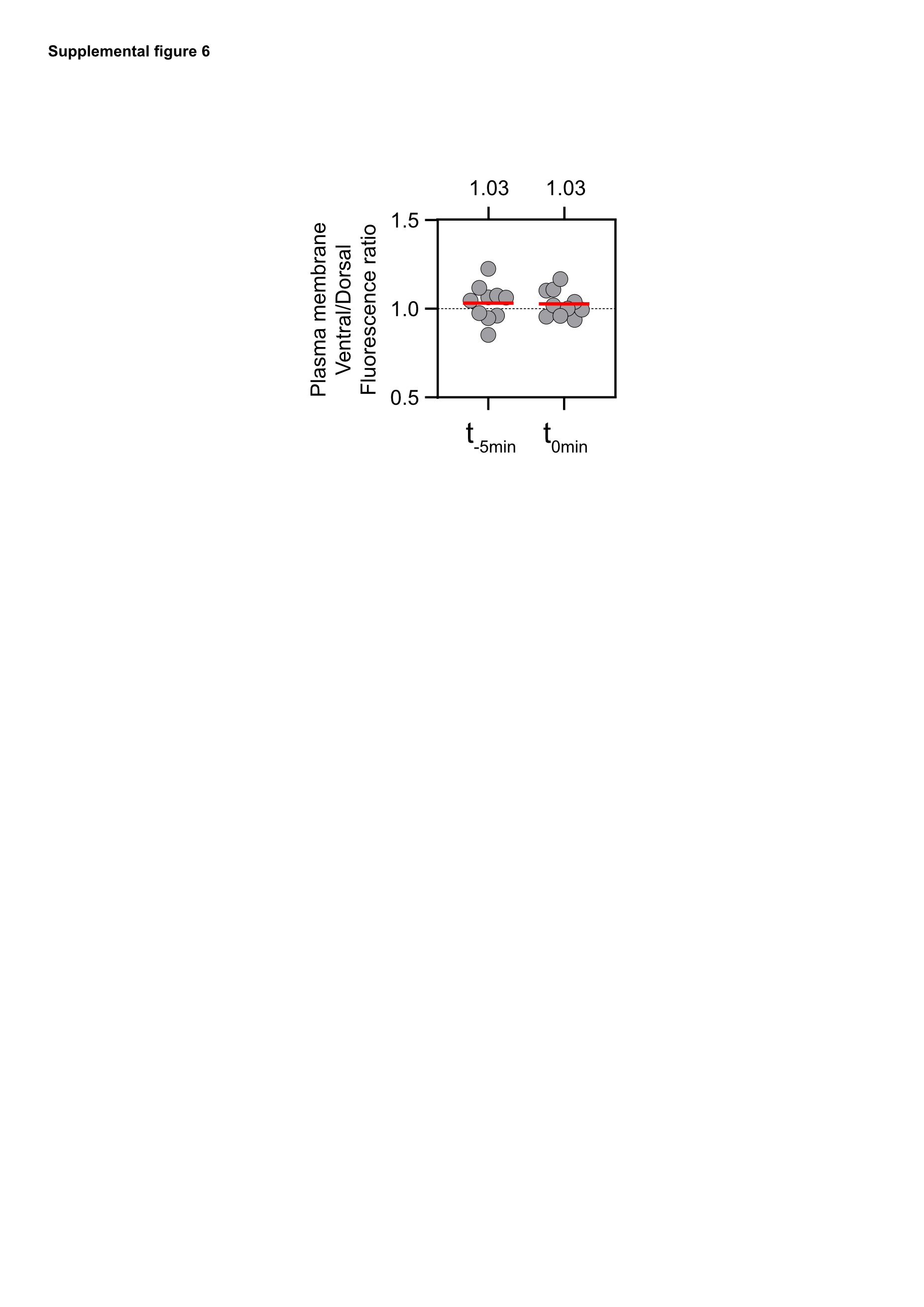

Supplement: S6 Fig — Each gray dot represents the ventral/dorsal fluorescence intensity ratio of 1 NSM neuroblast (n = 12). The mean values are indicated by the horizontal red lines and are also given on top. The horizontal black dotted line represents a fluorescence intensity ratio of 1, which indicates no asymmetry in fluorescence intensity between the ventral and dorsal side of the NSM neuroblast. (TIF) [file pbio.3001786.s006.tif]

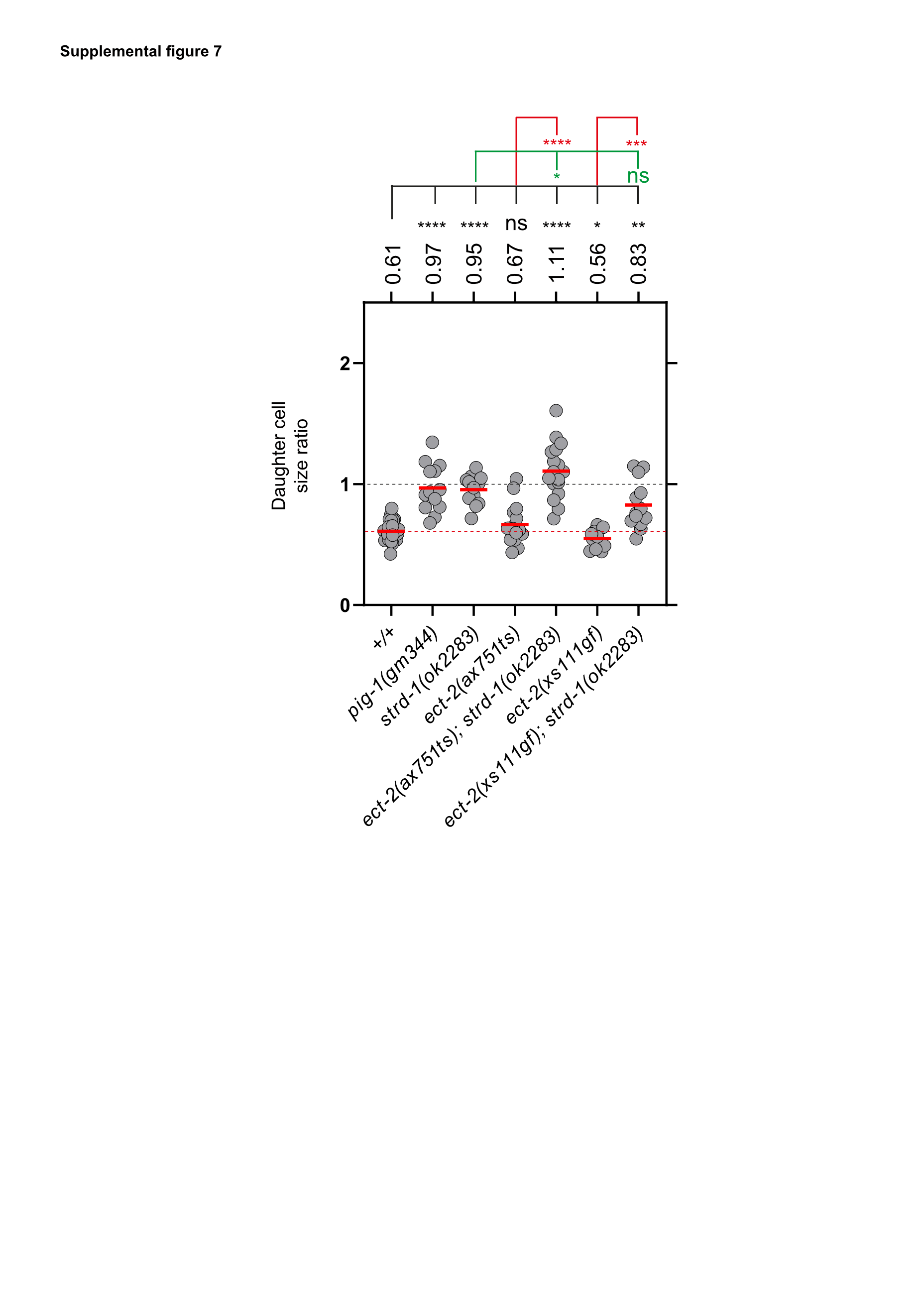

Supplement: S7 Fig — Each gray dot represents the daughter cell size ratio of 1 pair of daughter cells. The mean values are indicated using the horizontal red lines and are also provided on top. The horizontal red dotted line represents the mean daughter cell size ratio of wild-type (+/+) embryos for comparison. The horizontal black dotted line represents a daughter cell size ratio of 1.0 indicating equal division. Statistical significance was determined using the Welch’s 2 sample t test (**** = P < 0.0001, *** = P < 0.001, ** = P < 0.01, * = P < 0.05, ns = P > 0.05). (TIF) [file pbio.3001786.s007.tif]
