# Supplementary material for: A caspase–RhoGEF axis contributes to the cell size threshold for apoptotic death in developing Caenorhabditis elegans
Source: PLoS Biol. 2022 Oct 6;20(10):e3001786. doi: 10.1371/journal.pbio.3001786 (PMC9536578; doi:10.1371/journal.pbio.3001786)
Supplement: Attachment — Submitted filename: Rebuttal_26July2022.docx [file pbio.3001786.s008.docx]

**PBIOLOGY-D-22-00984R1**

“Functional interactions between the apoptosis pathway and cell size are coordinated by the *ced-3* caspase – *ect-2* RhoGEF axis”

**Rev. 1: W. Brent Derry – Note that this reviewer has signed his review**

The manuscript by Sethi et al. demonstrates a molecular connection between the C. elegans caspase CED-3 and the Rho guanine nucleotide exchange factor ECT-2 in establishing a cell size threshold for apoptotic death during embryonic development. Interestingly, this interaction does not result in the proteolytic processing of ECT-2. It is well established that somatic cells fated to die by apoptosis during C. elegans development are smaller than their surviving sister cells, but whether this is physiologically relevant has remained mysterious. Work from Dr.Conradt's lab previously showed mutants that cause symmetrical divisions of cells in lineages fated to die can suppress apoptosis, suggesting that a size threshold dictates life and death decisions. In this manuscript the authors present evidence that ECT-2 acts downstream of CED-3 to regulate asymmetric divisions, but upstream of CED-3 to promote apoptosis of the NSMsc and QL.pp neuroblasts. Overall, this is a solid manuscript and I only have a few suggestions/questions to help improve this excellent work.

Sethi et al: We thank the reviewer for their constructive comments and suggestions!

1. The authors show that recombinant ECT-2 co-purifies with recombinant CED-3 (or proCED-3). Does this interaction dependent on whether ECT-2 is GDP or GTP bound? This is important to know because it would help determine if a signal that activates ECT-2 (to its GTP-bound form) is triggers a physical interaction with CED-3.

Sethi et al: At least to our knowledge, it is the GTPase Rho that exists in a GDP- or GTP-bound state and not the RhoGEF ECT-2.

2. It is interesting that the Pleckstrin homology (PH) domain of ECT-2 binds CED-3. Normally, the PH domain interacts with phosphatidylinositol lipids. Have the authors checked if altering the phosphatidylinositol lipid content (for example, using an age-1 mutant) affects apoptosis and/or asymmetric divisions of the NSM or the QL lineage?

Sethi et al: This is an interesting suggestion. ect-2 is an essential gene involved in a number of processes during embryogenesis including cytokinesis, asymmetric protein localization and cell fate determination. Active ECT-2 protein localises to the cell cortex, and this is presumably mediated through the PH domain. To analyse age-1 mutants for defects in asymmetric cell division in cell death lineages is an interesting suggestion, and we will consider following this up in the future.

3. Given that CED-3 physically interacts with ECT-2 but does not cleave it, I wonder if this interaction affects the catalytic activity of CED-3? For example, does it stimulate or inhibit cleavage of CED-9?

Sethi et al: This is an excellent suggestion; however, CED-3-dependent cleavage of CED-9 has been proposed to occur in the daughter programmed to die whereas the interaction between CED-3 and ECT-2 that we describe occurs in mothers of cells programmed to die. Furthermore, to determine the effect of ECT-2 binding to CED-3 on CED-3 caspase activity, it will require the development of more quantitative and more sensitive assays for both the interaction between CED-3 and ECT-2 and CED-3-dependent cleavage of CED-9. We believe that these biochemical studies are beyond the scope of this manuscript.

4. Since cells normally fated to die by apoptosis often survive when they are a larger size (but not always) I am curious if the authors have considered that a pro-apoptotic factor such as EGL-1 or a microRNA specific for ced-9 is being diluted to prevent engagement of the apoptotic machinery? Can the authors estimate how the increased cell volume would affect the concentrations of these factors?

Sethi et al: How cell size impacts the activation or activity of the apoptosis pathway is a critical question and our future goal is to address exactly that question. Indeed, we plan to look at all components of the apoptosis pathway, including EGL-1 and CED-9. However, this is an entire new study. We believe that this is beyond the scope of the current manuscript.

5. The authors suggest that ECT-2 might engage proteins other than RHOA/ROCK/NMY to regulate apoptosis of neuroblasts. Have they considered the RAS/MAPK pathway since ECT-2 has been shown to regulate this pathway during vulva development (PMID: 16270101)?

Sethi et al: The reviewer raises another interesting point. While we do not consider it likely that ect-2 acts upstream of the Ras/MAPK pathway in mothers of cells programmed to die, we are interested in identifying signalling pathways that polarise these mother cells. The Ras/MAPK pathway is a candidate pathway, and we plan to investigate its potential role in the future.

Furthermore, the RAS/MAPK pathway also regulates developmental apoptosis (PMID: 11333233, 25144461). Although, it seems to have no role on NSMsc apoptosis (PMID:25144461), it might have a role in the QL.pp lineage.

Sethi et al: In PMID: 11333233, a large deletion that removes many loci including the let-60 RAS locus is described as impacting developmental apoptosis; however, at least to our knowledge, there was no follow-up study that confirmed a role specifically of let-60 RAS. In PMID: 25144461, the authors propose that the RAS/MAPK pathway impacts developmental apoptosis by affecting the transcriptional regulation of the egl-1 gene. As the reviewer states, the effect was found to be cell death lineage specific and to not affect the NSM lineage. For this reason, we are currently not considering the RAS/MAPK pathway as high priority.

6. The authors propose that ect-2 acts downstream of ced-3 to promote asymmetric cell division, but upstream of ced-3 to promote apoptosis. This is a bit confusing as written. To make this point clearer I suggest they revise the model in Figure 7 to better reflect these activities.

Sethi et al: We thank the reviewer for this suggestion. Based on this, we have modified the model in Figure 7 and added a new part to it, Part C.

7. The discussion is very long and should be shortened to be more concise.

Sethi et al: We have revised the discussion and shortened it.

**Rev. 2:** In this manuscript from Sethi et al, the authors build on their previous work showing that in C elegans division of programmed to die mothers of cells die unevenly due to a short burst of polar cortical contractility of the actomyosin network directly before division. This is due to a very low, sub-lethal, level of caspase activity in these cells, and is dependent on BH3-only-, APAF- and caspase-like genes.In this study, the authors convincingly show using Y2H and follow-up biochemical approaches that ECT-2 andced-3 interact at the plasma membrane at the dorsal side in smaller cells, permitting an accumulation of ECT-2 on the ventral side, importantly uncovering a new, non-apoptotic role for ced-3. In general, the experiments are very well performed, well controlled and contain the appropriate statistics. Overall, this is a very nice manuscript.However, for me, there is one question which is left unresolved, which if possible could be addressed further: why are larger cells less likely to undergo cell death? I assume this is due to the smaller effect sub-lethal caspase activity in these cells, but at least some more speculation in the discussion or some more experiments would be interesting to find out, such as addressing the question the authors themselves propose around APAF-1 dimerization.

Sethi et al: As the reviewer states, why larger cells are less likely to die is a critical question and as mentioned in our response to reviewer 1, we plan to address this question in the future. However, we believe that this is an entirely new study, which is beyond the scope of the manuscript. However, we have modified the discussion and added some more speculation on what the underlying mechanism(s) could be on page 22, 23 of the manuscript:

“A decrease in cell size could lead to an increase in concentration of critical pro-apoptotic factors, such as EGL-1 BH3-only. It may also facilitate the assembly of CED-4 Apaf-1 dimers into functional apoptosomes and thereby promote proCED-3 caspase maturation and activation. Future work will be needed to address this important question.”

We thank the reviewer for their constructive comments and suggestions!
